# Transition Metal Aluminum Boride as a New Candidate for Ambient-Condition Electrochemical Ammonia Synthesis

**DOI:** 10.1007/s40820-020-0400-z

**Published:** 2020-02-28

**Authors:** Yang Fu, Peter Richardson, Kangkang Li, Hai Yu, Bing Yu, Scott Donne, Erich Kisi, Tianyi Ma

**Affiliations:** 1 Discipline of Chemistry, School of Environmental and Life Sciences, University of Newcastle, Callaghan, NSW 2308 Australia; 2 School of Engineering, University of Newcastle, Callaghan, NSW 2308 Australia; 3 CSIRO Energy, 10 Murray Dwyer Circuit, Mayfield West, NSW 2304 Australia; 4 School of Environmental Science and Engineering, Fujian Normal University, Fuzhou, 350007 Fujian People’s Republic of China

**Keywords:** MAB phase, N_2_ reduction reaction, Electrocatalysis, Nanostructure

## Abstract

Achieving more meaningful N_2_ conversion by reducing the energy input and carbon footprint is now being investigated through a method of N_2_ fixation instead of the Haber–Bosch process. Unfortunately, the electrochemical N_2_ reduction reaction (NRR) method as a rising approach currently still shows low selectivity (Faradaic efficiency < 10%) and high-energy consumption [applied potential at least − 0.2 V versus the reversible hydrogen electrode (RHE)]. Here, the role of molybdenum aluminum boride single crystals, belonging to a family of ternary transition metal aluminum borides known as MAB phases, is reported for the electrochemical NRR for the first time, at a low applied potential (− 0.05 V versus RHE) under ambient conditions and in alkaline media. Due to the unique nano-laminated crystal structure of the MAB phase, these inexpensive materials have been found to exhibit excellent electrocatalytic performances (NH_3_ yield: 9.2 µg h^−1^ cm^−2^ mg_cat._^−1^, Faradaic efficiency: 30.1%) at the low overpotential, and to display a high chemical stability and sustained catalytic performance. In conjunction, further mechanism studies indicate B and Al as main-group metals show a highly selective affinity to N_2_ due to the strong interaction between the B 2*p*/Al 3*p* band and the N 2*p* orbitals, while Mo exhibits specific catalytic activity toward the subsequent reduction reaction. Overall, the MAB-phase catalyst under the synergy of the elements within ternary compound can suppress the hydrogen evolution reaction and achieve enhanced NRR performance. The significance of this work is to provide a promising candidate in the future synthesis of ammonia.

**Electronic supplementary material:**

The online version of this article (10.1007/s40820-020-0400-z) contains supplementary material, which is available to authorized users.

## Highlights

- Molybdenum aluminum boride single crystals as layered ternary borides were firstly applied for the electrochemical N_2_ reduction reaction under ambient conditions and in alkaline media, displaying excellent electrocatalytic performances at the low overpotential.
- Through the combination of the strong interaction of Al/B band and N orbitals and the special crystal structure exposing more active sites, synergistic effect of the elements was verified to achieve the enhancement of N_2_ reduction reaction process and the limitation of hydrogen evolution reaction.

**Electronic supplementary material:**

The online version of this article (10.1007/s40820-020-0400-z) contains supplementary material, which is available to authorized users.

## Graphical abstract

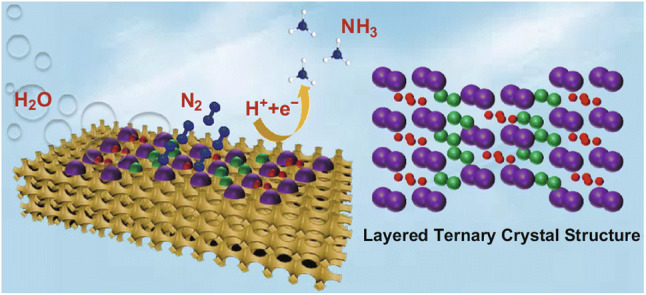

## Introduction

Ammonia (NH_3_) is not only an important chemical in industrial production, including pharmaceutical, synthetic fibers and fertilizer production, but also an energy conversion carrier, such as being an ideal storage medium for hydrogen (H_2_) [1–4]. Additionally, it is the only currently known carbon-free energy carrier that does not release carbon dioxide (CO_2_) emissions [4]. However, as the most abundant molecule in the atmosphere, nitrogen (N_2_) is extremely difficult to be converted into NH_3_ due to its strong bond energy, low polarizability and lack of a dipole moment [5]. At present, ammonia is mainly produced by the Haber–Bosch process at high temperature and pressure that reduces N_2_ to NH_3_ within coal-based or natural gas-based ammonia plants [6]. However, the harsh reaction conditions and the use of natural gas as the hydrogen source lead to large energy consumption and serious greenhouse gas emission [7–10]. Therefore, it is of great significance to design and develop a sustainable and environmentally benign approach for NH_3_ synthesis.

Recently, the electrocatalytic N_2_ reduction reaction (NRR) using aqueous electrolytes for synthesizing ammonia at ambient conditions has attracted intensive research interest [5, 11, 12]. Motivated by the impressive advantages, including mild conditions supporting the feasibility to reduce the energy input and cutting down the carbon footprint, and the simple reactor designs that outweigh the complexity of ammonia production plants [12], the electrocatalytic NRR under an ambient condition has achieved considerable progress since 2016 [11]. Until now, various nanomaterials have been reported as potential catalysts for NRR electrocatalysis, including noble metals (Au, Ru, Pd, Pt, Ag, etc.), transition metals (Fe, Ti, Mo, Cr, Co, etc.) and their oxides, carbides, nitrides and sulfides, metal-free materials (B, C, N, S, P, etc.), and their relevant composites. Development of these materials, coupled with some effective strategies to improve the catalytic performance including defect engineering, interface engineering, electrolyte manipulation and cell design, has been with the goal of improving NH_3_ yield and Faradaic efficiency (FE) [13–21]. Despite its progress in a short time, this research field is undoubtedly in its infancy and faces many problems. Firstly, most catalysts show a higher overpotential for the electrochemical NRR than for the hydrogen evolution reaction (HER) [22, 23]. Therefore, most published research findings exhibited limited selectivity and activity in aqueous solutions due to the strong HER competition [17, 24–32]. Secondly, previous reports indicated that non-aqueous solutions or hydrophobic catalysts could suppress HER by limiting proton concentration [33, 34]. However, the lack of proton supply would also limit activity. Hence, electrocatalysts that selectively and efficiently reduce nitrogen to ammonia remain elusive. Thirdly, the amount of ammonia produced by the electrochemical NRR method is usually so small that it is difficult to attribute it solely to electrochemical nitrogen fixation and exclude contamination [35]. There are various possible sources of ammonia: On the one hand, it can be present in air, human breath or ion-exchange membranes [25]; on the other hand, it can be generated from labile nitrogen-containing compounds (for example, nitrates, amines, nitrites and nitrogen oxides) that are typically present in the nitrogen gas stream [26], in the atmosphere or even in the catalyst itself [36]. Besides, N_2_ gas shows low solubility in water so the amount of N_2_ actually involved in the NRR is very small. Additionally, the average catalyst loading is less than 1 mg cm^−2^, which limits the total current density to less than 10 mA cm^−2^ and NRR partial current density to as low as ~ 0.1 mA cm^−2^ [11]. Therefore, these limitations collectively result in the inferior yield and selectivity of ammonia in the electrocatalyzed NRR process.

An alternative strategy for achieving a high surface area uses “multicomponent” materials, in which different parts of the structure can behave as the active catalytic sites and the inert HER competitive sites. Such architectures allow for the implementation of the active site separation concept, which has been shown to be effective in a number of catalysts [37], for example, MAX phases and MXenes [38, 39]. MAX phases, as nanolayered ternary compounds, comprise a large family of M_*n*+1_AX_*n*_ materials where typically M is an early transition metal, A is an A group element (for example, Al or Si), X is carbon or nitrogen and *n* = 1–3 [40, 41]. MXenes, a novel family of 2D metal carbides and nitrides, can be derived by chemically etching and exfoliating MAX phases [42].

Similarly, MAB phases, first named in 2015 [43], are structurally similar to MAX phases, which have received increasing attention due to their combination of ceramic and metallic material properties: high flexural strength, compressive strength, oxidation resistance, metallic conductivity and high thermal conductivity [41, 44]. Meanwhile, these MAB phases, as electrocatalytic materials, have attracted our attention due to their atomically layered crystal structure of ternary compound. MoAlB crystallises in the orthorhombic *cmcm* space group and is arranged as slabs of trigonal prismatic Mo_6_B ceramics, which are the orthorhombic β-MoB phase, interleaved with two metallic planes of Al atoms. Additionally, the two-dimensional derivative of MAX phases, MXenes, has shown great promise for a large range of chemical processing applications including hydrogen and oxygen evolution catalysts, electro-storage devices and environmental adsorbants [45–47], which inspires us to explore the possibility of similar electrochemical properties of MAB phases. For example, Ma et al. [48] designed a hybrid film of overlapped g-C_3_N_4_ and Ti_3_C_2_ (MXene) nanosheets as a highly efficient oxygen electrode. The hybrid film through Ti–N_*x*_ interaction, forming a porous free-standing film with hydrophilic surface and conductive framework, exhibits excellent performance in catalyzing the oxygen evolution reaction (OER). Further, due to oxygen terminations on the basal plane providing catalytic active sites, Jiang et al. [49] reported a method to significantly improve the HER performance of Ti_3_C_2_ MXene by modifying terminations of MXenes on the basal. This has been confirmed for the Fe_2_AlB_2_ and MoAlB as MAB phases or their two-dimensional derivatives, which were found to play a part in the oxygen and hydrogen evolution processes [37, 44].

Herein, the behavior of MoAlB single crystals (SCs), as a new type of NRR catalysts based on the transition metal aluminum boride phase (MAB phase) family, is reported for the first time. In brief, the MoAlB SCs were supported on a free-standing copper foam (Cu foam) to make an electrode for electrocatalytic NRR in alkaline electrolytes under ambient conditions. In the electrocatalytic NRR system, due to the strong interactions between Al/B atoms and nitrogen atoms, the competitive HER was largely suppressed. In this work, the MoAlB SCs exhibited high NRR activity and selectivity at a low applied potential at room temperature and ambient pressure in a 0.1 M KOH electrolyte. The catalysts are able to be prepared with low cost due to the high abundance and low price of the starting materials. These results are superior to most reported catalysts and distinguish MoAlB SCs as a promising catalyst in electrochemical NRR applications.

## Experimental

### Material and Chemicals

Mo powder (Metco), B powder (Sigma-Aldrich, > 95%, < 1 µm), Al powder (Australian Metal Powders Supplies, > 99%, 45 µm), Nafion^®^ perfluorinated resin solution (Sigma-Aldrich, 5 wt%), Nafion^®^ 117 membrane, potassium hydroxide (Sigma-Aldrich, 90%), low-level ammonia pH adjusting ISA (Thermo SCIENTIFIC), ammonia standard solution (Thermo SCIENTIFIC), phenol (BDH Laboratory Supplies), ammonium sulfate (BDH Chemical, Australia Pty. Ltd.), ethanol (Merck KGaA), sodium nitroferricyanide (III) dehydrate (Sigma-Aldrich, > 99%), trisodium citrate dehydrate (Sigma-Aldrich), sodium hydroxide (Sigma-Aldrich), sodium hypochlorite solution (Sigma-Aldrich) and deionized water (Millipore, 18.2 MΩ cm) was used as the solvent, N_2_ gas (99.99%) and Ar gas (99.99%). All chemical regents were used as received without further purification.

### Preparation of the Membrane Electrode

#### Electrocatalysts Synthesis

Bulk MoAlB powders were prepared using the following procedure. Mo, B and Al powders were mixed with a molar ratio of Mo:Al:B = 1:1.3:1. The powder mixture was cold pressed to 220 MPa in a 15-mm-diameter steel die. The pellet was placed in an alumina crucible and heated in a tube furnace under flowing argon to 1200 °C at 5 °C min^−1^ and held for 2 h before cooling to room temperature at 5 °C min^−1^. The reacted sample was crushed into < 45 µm powder, placed in a 12.7-mm-diameter graphite foil-lined graphite die, and hot pressed to further react intermediate MoB and Mo–Al phases. The die and sample were heated in a hot-press furnace under flowing argon to 1400 °C at 10 °C min^−1^ and held for 30 min. Pressure was applied gradually from 800 °C to a maximum of 50 MPa at 1400 °C. The surface of the solid MoAlB sample was ground to remove graphite, then was mechanically crushed and sieved to < 45 µm particle size.

**Scheme 1:**
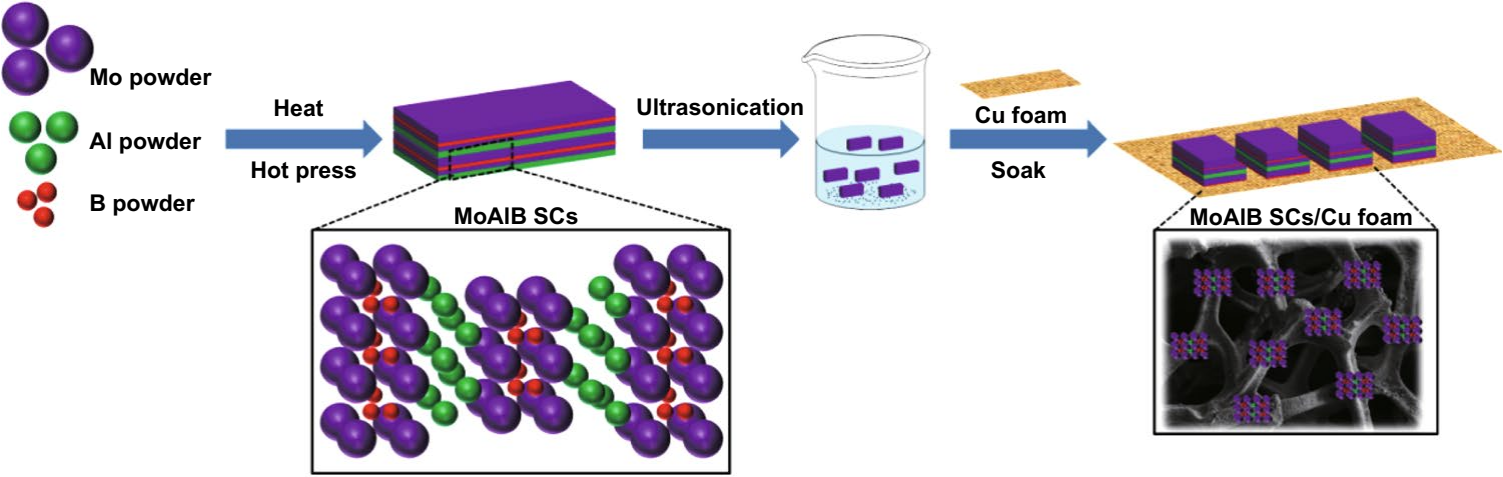
Illustration of the synthesis process of MoAlB SCs and MoAlB SCs/Cu foam electrode

MoAlB single crystals (SCs) were prepared using a modification of a reported procedure [50]. As shown in Scheme 1, the samples were prepared by first synthesizing MoB powder by mixing Mo and B powders in a stoichiometric ratio (Mo:B = 1:1). The powder mixture was cold pressed to 220 MPa in a 15-mm-diameter steel die. The pellet was placed in an alumina crucible and heated in a tube furnace under flowing argon to 1200 °C at 5 °C min^−1^ and held for 2 h before cooling to room temperature at 5 °C min^−1^. The reacted MoB powder was crushed and mixed with Al powder with a molar ratio of MoB:Al = 1:1.3. The pellet was placed in an alumina crucible and heated in a tube furnace under flowing argon to 1000 °C at 5 °C min^−1^ and held for 15 h before cooling to room temperature at 5 °C min^−1^. The loosely sintered products were carefully crushed into powder by mortar and pestle and placed to obtain the MoAlB SCs. Finally, the cleaned copper foam was immersed in a MoAlB SC ink with the aid of a Nafion binder to obtain a MoAlB SC/Cu foam. (Details of the electrode preparation can be found in Sect. 2.2.2).

#### Preparation of the Electrode

*Catalyst ink* 0.25 g of the catalyst material was suspended in 9 mL deionized water and 1 mL of 5 wt% Nafion^®^ solution, which is predominantly water, was added. Hence, the catalyst material formed 25 mg mL^−1^ of the ink. The solution was then ultrasonicated for 1 h in an attempt to break down any agglomerated particles and aggregates to as small as possible to obtain a uniform solution.

*Pretreatment of electrode* The 1-cm^2^ copper foams were ultrasonicated in 0.1 M HCl, deionized water and acetone, respectively. Then the electrodes were placed in an oven to dry. After the pretreatment, the electrodes were dipped in the above ink three times. After each dip, it was ensured that the 1-cm^2^ electrode was completely covered in the ink. The coated electrode was then placed in an oven at 100 °C for 5 min. The same coating process was then repeated a further two times. In the end, the cleaned copper foam was immersed in the catalyst ink with the aid of a Nafion binder to obtain the electrode.

#### Proton Exchange Membrane Pretreatment

A Nafion^®^ 117 membrane was cut into small pieces and then treated with 3 wt% H_2_O_2_ water solution, deionized water, 1 mol L^−1^ H_2_SO_4_ and deionized water for 1 h at 80 °C, respectively. Finally, the obtained membrane was repeatedly rinsed until neutral pH was obtained and then was preserved in deionized water.

### Electrochemical Measurements

All electrochemical measurements were carried out on a CHI760e electrochemical station at 20 °C. Electrochemical measurements were carried out on a three-electrode system with Pt wire as the counter electrode, Ag/AgCl (3.5 M KCl) as the reference electrode and modified copper foam as the working electrode. The gas-tight two-compartment electrochemical cell was separated by a piece of Nafion^®^ 117 membrane at room temperature. 250 mL min^−1^ of N_2_ (99.99%) was introduced to the cathode portion of the system from 30 min beforehand, until the end of the reaction. All of the potentials in this work were calculated to a reversible hydrogen electrode (RHE) scale based on the Nernst equation (ERHE = *E*_Ag/AgCl_ + 0.059 × pH + 0.2046). The value of 0.2046 depended on the KCl concentration in the reference electrode. (Details of detection of ammonia can be found in the supporting information (SI)).

## Results and Discussion

### Materials Characterization

**Fig. 1:**
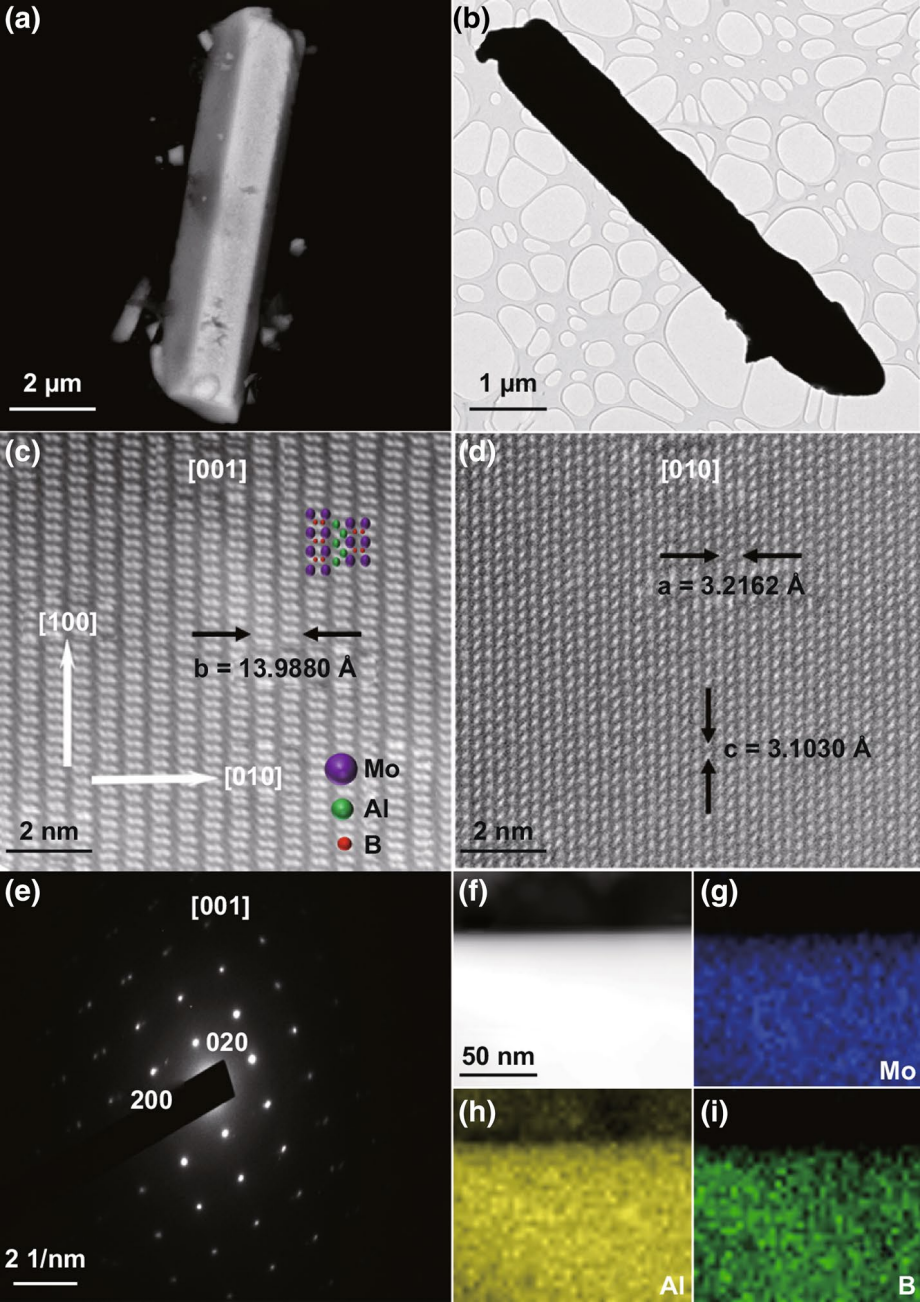
**a** A typical SEM image and **b** TEM image of a MoAlB SC. **c** HAADF-STEM image of a MoAlB SC [001] and crystal structures of representative MoAlB (top left). **d** HAADF-STEM image of a MoAlB SC [010]. **e** HRTEM corresponding SAED pattern of a MoAlB SC. **f–i** HAADF-STEM image and corresponding EDS elemental mapping results for a MoAlB SC

The nano-/microstructure of as-prepared MoAlB SCs was examined by scanning electron microscopy (SEM). Figures 1a and S7 show the rod-like MoAlB SCs formed at 1000 °C, which have an average length of approximately 10 μm. Furthermore, the observed morphology of MoAlB SCs by SEM is consistent with that identified by transmission electron microscopy (TEM) in Fig. 1b. The scanning electron microscopy energy-dispersive X-ray spectroscopy (SEM–EDS) analysis indicates an even distribution of Mo and Al co-localization in the crystals and confirms the expected 1:1 Mo:Al ratio (Fig. S8 and Table S1). The high-angle annular dark-field scanning transmission electron microscopy (HAADF-STEM) images in Fig. 1c, d focus on regions within a few micrometers of the crystal surface. The images were collected in the [001] and [010] crystallographic directions. Corresponding 2D structural models along the same zone axis, shown as colored insets in Fig. 1c, are compared to the contrast image and show good agreement. This supports the hypothesized formation of the layered ternary borides as a result of a stepwise intercalation of Al into MoB during the formation of MoAlB. Additionally, the contrast image clearly shows the atomic sequence of the crystal structures: an Al double layer distributes between the two adjacent MoB layers. The double layers of bright dots correspond to the Mo atoms while the gray dots in between correspond to the Al layers. Figure 1e exhibits the selected area electron diffraction (SAED) patterns of MoAlB SCs along the [001] zone axis. The HAADF-STEM image and corresponding EDS elemental mapping images of MoAlB SCs are displayed in Fig. 1f–i. This analysis further verifies the homogeneous distribution of Mo, Al and B over the entire structure.

**Fig. 2:**
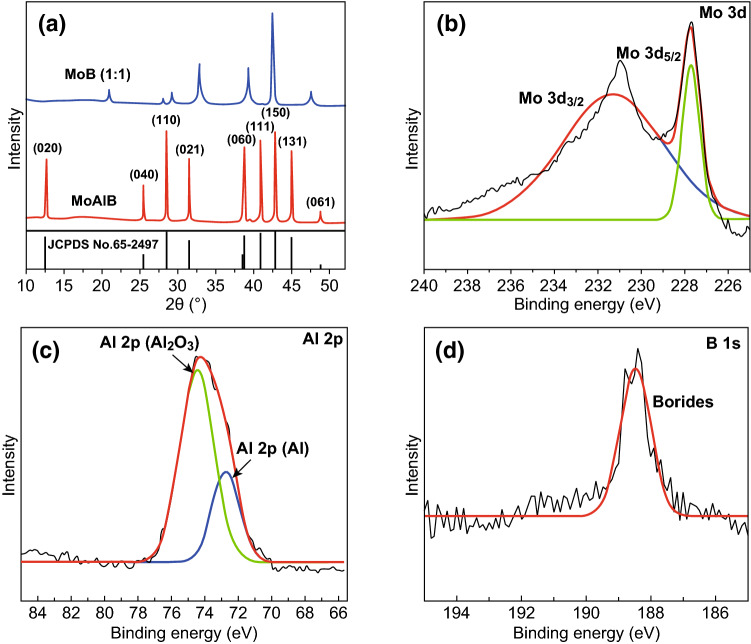
**a** XRD patterns for MoAlB SCs. **b** XPS spectra of Mo 3*d* electrons for MoAlB SCs. **c** XPS spectra of Al 2*p* for MoAlB SCs. **d** XPS spectra of B 1*s* electrons for MoAlB SCs

X-ray diffraction (XRD) was utilized to confirm the structure and composition of MoAlB and MoB (1:1) samples. As shown in Fig. 2a, the XRD pattern of MoAlB SCs shows characteristic diffraction peaks including (020), (040), (110), (021), (060), (111), (150), (131) and (061), which reveal a collection of single crystalline phase ground into a powder matching well with the simulated pattern for MoAlB (JCPDS No. 65-2497). Besides, analysis of the XRD data of the MoAlB powders shows that it is predominantly single phase (98.8 wt%) with a small amount of impurity phase of Al_2_O_3_ (1.2 wt%). This is likely due to oxidation of the Al metal in the presence of oxygen; the presence of oxygen may have been due to: impurity within the argon supply during synthesis, formation of a thin Al_2_O_3_ skin around the Al powder during storage of the material in air, or as an impurity in un-reacted boron contained within the MoB powder. The *a, b* and *c* lattice parameters for MoAlB were calculated to be 3.2126, 13.9880 and 3.1042 Å, respectively, from Rietveld refinement of the XRD data. These values are close to published data (*a* = 3.2162, *b* = 14.062 and *c* = 3.1030 Å) [43, 51], verifying the quality of this synthesis process. Figure S11 shows the XPS spectrum of MoAlB SCs, with four peaks appearing at 74.0, 232.0, 288.1 and 531.5 eV corresponding to the Al 2*p*, Mo 3*d*, C 1*s* and O 1*s* electrons, respectively. However, the presence of the B 1*s* electrons was not detected in the XPS analysis of the MoAlB SCs because B atoms are too light to be detected using this technique. Figure 2b provides a high-resolution XPS spectrum of the Mo 3*d* signal deconvoluted into two peaks located at 228.2 and 231.5 eV, which can be assigned to the Mo 3*d*_5/2_ and Mo 3*d*_3/2_ electrons of Mo in Mo–Al–B, respectively [52]. It is attributed to Mo atom bound with Al and B atom, respectively. Figure 2c shows the XPS spectrum of Al 2*p*, fit with two components: one for Al_2_O_3_, the other for Mo–Al–B. The binding energy peak of Mo–Al–B in the MoAlB SCs is at 73.0 eV and coincides with the peak of 73.0 eV obtained from our spectrum of elemental Al. The other peak at 74.9 eV corresponds with Al_2_O_3_. The presence of Al_2_O_3_ here is consistent with the analysis of the XRD data. Figure 2d shows the XPS spectrum of the B 1*s* with only one strong peak at 188.5 eV. This indicates that no boron oxide was detected on the MoAlB SCs sample and most likely the boron was fully reacted at this point. These collective data are indicative of the successful synthesis of MoAlB SCs.

### Electrochemical Nitrogen Reduction

**Fig. 3:**
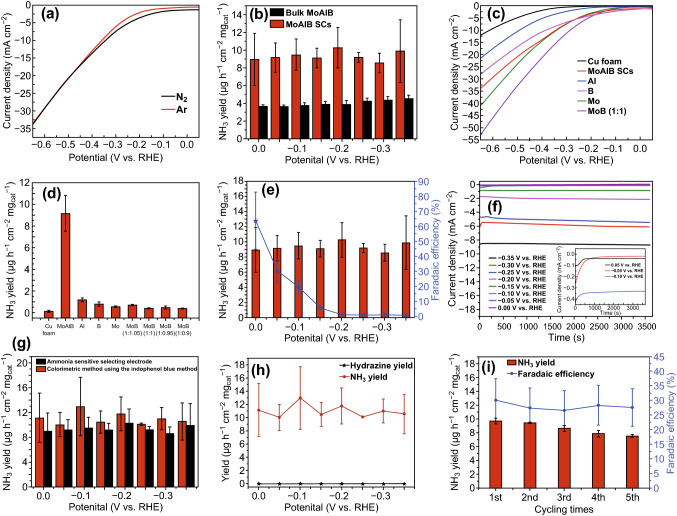
**a** LSV curves of MoAlB SCs/Cu foam electrode in N_2_- and Ar-saturated aqueous solutions of 0.1 M KOH. **b** Comparison of ammonia yield at different potentials ranging from 0 to − 0.35 V versus RHE between MoAlB SCs and bulk MoAlB. **c** LSV curves of pure Cu foam, MoAlB SCs, Al, B, Mo and MoB (1:1) electrodes in an N_2_-saturated aqueous solution of 0.1 M KOH. **d** Ammonia yield at − 0.05 V versus RHE for pure Cu foam, MoAlB SCs, Al, B, Mo and MoB (1:1) electrodes in an N_2_-saturated aqueous solution of 0.1 M KOH. **e** Faradaic efficiency and ammonia yield at different potentials ranging from 0 to − 0.35 V versus RHE for MoAlB SCs. **f** Chronoamperometry results at the corresponding potentials. **g** Comparison of the ammonia-sensitive selecting electrode and indophenol blue reagent-based colorimetric method for the quantitative analysis of ammonia yield. **h** Indophenol blue reagent-based colorimetric method for the quantitative analysis of ammonia yield and Watt and Chrisp method for the quantitative analysis of hydrazine yield. **i** Faradaic efficiency and ammonia yield during five consecutive cycles

To evaluate the electrocatalytic NRR activities of MoAlB SCs under ambient conditions, electrochemical tests were performed in N_2_-saturated 0.1 M KOH electrolyte, including linear sweep voltammetry (LSV), chronoamperometry and impedance. All tests were performed in a two-compartment cell separated by a proton-conductive cation exchange membrane (Nafion^®^ 117), in which the protons (H^+^) can react with N_2_ to form ammonia over the catalyst. At first, the LSV curves for MoAlB SCs in Ar- and N_2_-saturated 0.1 M KOH solutions were measured to verify the source of ammonia (Fig. 3a). In the Ar-saturated solution, the increase in current density after − 0.1 V versus RHE is caused by the HER, which competes with the NRR. In contrast, when the applied potential is more positive than − 0.4 V versus RHE, a clear reduction in the current density for the N_2_-saturated solution is observed compared with that of the Ar-saturated solution. This provides evidence that the catalytic reduction of N_2_ to NH_3_ does in fact take place in this system. When the applied potential was set more negative than − 0.4 V versus RHE, the current densities in N_2_-saturated and Ar-saturated solutions were very close, likely due to the dominant behavior of HER compared to the NRR in this system. In addition, for further confirmation of successful ammonia synthesis, the corresponding NH_3_ concentrations were measured by using the ammonia-selective electrode method for qualitative analysis of ammonia in the electrolyte after 1 h of electrolysis in the presence of continuous Ar and N_2_ bubbling (further details are provided in Fig. S13), which shows that the values of NH_3_ yield are derived from NH_3_ concentrations. In the Ar system, negligible ammonia was detected in the electrolyte due to background signal interference. These results demonstrate that the N sources for ammonia synthesis are exclusively provided by the N_2_ feed gas, indicating that the electrocatalytic N_2_ reduction can be realized by the as-prepared MoAlB SCs.

For evaluating the advantage of the single-crystal structure in the NRR process, two different samples with the same composition and different structures, bulk MoAlB (polycrystalline phase) and MoAlB SCs were synthesized by different methods. Compared to bulk MoAlB, as shown in Fig. 3b, the MoAlB SCs sample shows better electrochemical N_2_ reduction performance, which can be attributed to the uniform crystal orientation that exposes dominantly [010] facets [53]. Because the area of [0l0] plane was much larger than [l00] and [00l] plane [53]. Meanwhile, compared with MoAlB SCs, as shown in Fig. S9, bulk MoAlB is a polycrystalline structure and particles are a little larger in size. Furthermore, it is known that the catalytic performance is determined by the size of particles as a key role. One is due to increasing of specific activity per metal atom generally with decreasing size of the particles [54]. The second one is to expose more catalytic sites because of small size. Thus, a hypothesis is able to be presented that more active sites and higher specific activity per metal atom are provided by the MoAlB SCs for facilitating the NRR process.

Figure 3c shows the LSV curves of pure Cu foam, MoAlB SCs/Cu foam, Al/Cu foam, B/Cu foam, Mo/Cu foam and MoB (1:1)/Cu foam for electrocatalytic NRR. At all applied potentials, pure Cu foam had a much lower current density onset potential than that of others, which can be attributed to the inert HER activity of pure Cu foam. In addition, as shown in Fig. 3d, ammonia yields of pure Cu foam, MoAlB SCs/Cu foam, Al/Cu foam, B/Cu foam, Mo/Cu foam and MoB (1:1)/Cu foam were obtained at a low applied potential (− 0.05 V versus RHE); all of these samples, with the exception of MoAlB SCs, exhibit almost no ammonia detected at this potential. The results demonstrate that the individual elements (Mo, Al and B) show a poor NRR activity. Meanwhile, comparing MoB samples at the different ratios, it is found that changing the relative amount of Mo and B has little influence on the overall activity. These results further confirm that only MoAlB SCs within the Mo–Al–B system possess activity toward the electrocatalytic NRR. Therefore, it is probably inferred here that the Mo, Al and B elements along with the unique structure of the MoAlB SCs could play a synergistic role in the electrocatalytic NRR.

Figure 3e shows the Faradaic efficiencies (FEs) and ammonia yields of MoAlB SCs under various applied potentials ranging from 0.0 to − 0.35 V versus RHE. The data in this figure were obtained based on the ammonia-selective electrode method. As observed in Fig. 3e, ammonia yields under various applied potentials show no obvious difference. However, FEs experience a gradual decreasing trend as the applied potential is shifted from 0.0 to − 0.35 V. In fact, as shown in Fig. 3f, a remarkable increase in the current density is observed with the increase in applied potentials. A likely explanation is that, due to the dominance of HER at higher overpotentials, the surface of MoAlB SCs was mainly occupied by evolving hydrogen molecules which would block the mass transfer of N_2_ to the surface of MoAlB SCs. This limits the electrocatalytic NRR activity [55] and results in the decline of the FEs.

In addition, to confirm the reliability of the ammonia-selective electrode method for ammonia detection, it was compared with a colorimetric method using an indophenol blue reagent, which gave consistent results (Fig. 3g). It is also found that the NH_3_ yield values determined by the colorimetric method are slightly higher than those determined by the ammonia-selective electrode method, possibly due to contaminants (metal residues, etc.) [56]. In conjunction, it has been reported that the determination of ammonia-selective electrode is not interfered by the contaminants [57]. Because N_2_H_4_ is a possible by-product during the electrocatalytic N_2_ reduction process, the colorimetric method was also used to determine whether any N_2_H_4_ was produced. No N_2_H_4_ is detected in the electrolyte after 1 h of electrolysis in the presence of continuous N_2_ bubbling (Fig. 3h), indicating that the as-prepared MoAlB SCs electrode has good selectivity for the NRR.

In Fig. 3e, MoAlB SCs exhibit higher FEs at low overpotentials. Although the highest FE was 63.7% at 0.0 V versus RHE, the value has low credibility due to a large relative error value resulting from a very low current density. Therefore, in all comparative experiments, an applied potential of − 0.05 V versus RHE was determined to be the most appropriate and was used. The NH_3_ yield, which was normalized based on the weight of the catalysts, and FEs of MoAlB SCs at − 0.05 V versus RHE are 9.2 µg h^−1^ cm^−2^ mg_cat._^−1^ and 30.1%, respectively (Fig. 3e). As far as we know, the NH_3_ yields and FEs that the MoAlB SCs achieved at a low applied potential are comparable to recently reported NRR electrocatalysts (Table S2). In this work, an ultralow applied potential (− 0.05 V versus RHE), closed to theory potential, is used for MoAlB SCs, making it one of the most active and selective electrocatalyst candidates for future NRR research at ambient conditions.

The stability of the MoAlB SCs for electrocatalytic N_2_ reduction was evaluated by consecutive recycling electrolysis at 0.05 V versus RHE. The ammonia yield and current efficiency data contain no significant fluctuation during five consecutive cycles (Fig. 3i), indicating the high stability of MoAlB SCs for electrochemical N_2_ reduction. Additionally, the stability of MoAlB SCs was also assessed by scanning at a constant potential of − 0.05 versus RHE for 10 h. The current density presented no obvious changes (Fig. S14), further indicating that MoAlB SCs can effectively produce NH_3_ over a long period of time. Besides, morphologies and elemental analysis of MoAlB SCs/Cu foam electrode before and after NRR stability tests were characterized by SEM. The SEM images present rod-like morphology, which have no obvious changes (Fig. S15). As shown in Fig. S15 and Table S3, except potassium element observed in the electrode after NRR stability tests, other element species and the corresponding amounts of atoms in EDS region scan analysis look almost the same. The fluorine is from Nafion^®^ solution and potassium is from KOH electrolyte. Therefore, these results confirm that this nanolayered ternary boride has an excellent chemical stability chemical structure during the NRR process.

### Mechanistic Study

**Fig. 4:**
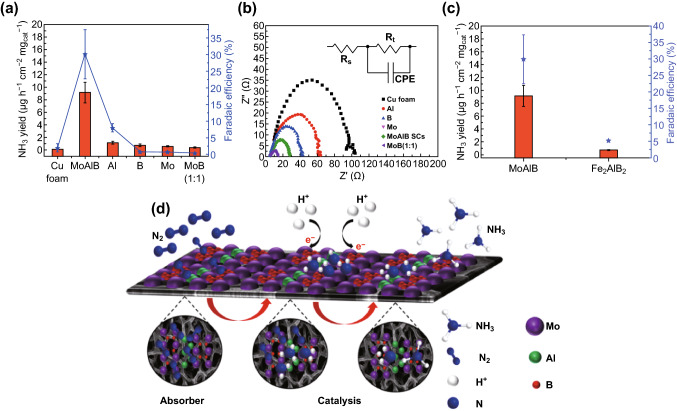
**a** Comparison of ammonia yield and Faradaic efficiencies at − 0.05 V versus RHE for pure Cu foam, MoAlB SCs, Al, B, Mo and MoB (1: 1) electrodes in an N_2_-saturated aqueous solution of 0.1 M KOH. **b** EIS (recorded at − 0.3 V versus RHE with inset showing the equivalent circuit diagram) of pure Cu foam, MoAlB SCs, Al, B, Mo and MoB (1: 1) in an N_2_-saturated aqueous solution of 0.1 M KOH. **c** Ammonia yield and Faradaic efficiencies at − 0.05 V versus RHE for MoAlB SCs and Fe_2_AlB_2_. **d** Mechanism of electrochemical NRR based on MoAlB SCs

To evaluate the electrocatalytic NRR mechanism of MoAlB SCs under ambient conditions, electrochemical comparison tests were performed. Firstly, as shown in Fig. 4a, compared to MoAlB SCs, pure Cu foam, Al/Cu foam, B/Cu foam, Mo/Cu foam and MoB (1:1)/Cu foam specimens exhibit almost no ammonia detection at a low applied potential (− 0.05 V versus RHE). This confirms that they possess no electrocatalytic activity toward the NRR process. In conjunction, MoAlB SCs and Al metal show higher FE values than the other compared specimens, which is attributed to the suppression of the HER process. Additionally, this is also confirmed by electrochemical impedance spectroscopy (EIS, Fig. 4b). The electron transfer resistance (*R*_t_) at the electrode surface is derived from the semicircle domains of impedance spectra, which is used to describe the interface properties of the electrode. The semicircle diameter of MoAlB SCs is much smaller than that of the control group of catalysts [Mo and MoB (1: 1)]. However, diameters for the B, Al and pure Cu foam are much smaller than that of MoAlB SCs. On the one hand, this is due to the lower contact and charge transfer impedance in MoAlB SCs consisting of Al and B. On the other hand, poor reactivities shown by B, Al and pure Cu foam indicate that less charge transfer is involved in the reaction, which is consistent with the data in Fig. 4a. In a previous report [58], it has already been verified that main-group metals (p metals) can exhibit much higher electrochemical NRR selectivity and activity than the intensively studied transition metals (d metals) due to the stronger interactions between the p orbitals of metal substrates and nitrogen absorbers. Meanwhile, to the best of our knowledge, most metals with theoretically high electrocatalytic NRR activity are transition metals, which exhibit very poor selectivity due to strong HER competition [22, 23]. Therefore, it is conceived that catalysts comprised of aluminum and boron may bind nitrogen more strongly than hydrogen and could exhibit higher NRR selectivity. However, because their binding to nitrogen is so strong and the desorption of certain intermediates would be very slow, their NRR activity could be limited [58]. Due to the critical step of N_2_ adsorption for the NRR process, the N_2_ adsorption behaviors of MoAlB SCs, Mo and MoB (1:1) were further evaluated by N_2_-TPD as shown in Fig. S16. Two adsorbed N_2_ peaks in as-prepared catalysts and only one adsorbed N_2_ peak in Mo are observed. The peak at about 150 °C is related to physical adsorption but not for Mo. The peak at 340 °C observed for MoAlB SCs, Mo and MoB (1:1) is related to the chemisorption species of N_2_. This result indicates that nitrogen vacancies could introduce many chemical adsorption sites on the surface of the catalysts. Because chemisorption is generally associated with activation, these chemical adsorption sites will activate N_2_ for nitrogen fixation [59]. Thus, the higher nitrogen vacancies concentration of MoAlB SCs causes the more chemical adsorption sites, leading to the higher NRR performance. Therefore, we propose that the Mo, Al and B elements in MoAlB SCs should play a synergistic role in the electrocatalytic NRR process.

To differentiate the catalytic site from the three elements in the system, Figs. S17 and 4c show that MoAlB SCs exhibit higher reduction current density and NH_3_ yield than a second MAB phase, Fe_2_AlB_2_. This compound is very structurally similar to MoAlB, except that only one Al plane interleaves the trigonal prismatic slabs rather than two. This indicates that Mo most likely plays a catalytic role rather than Al and B in the electrochemical NRR process. Thus, on the basis of the above discussion, the mechanism of electrochemical NRR based on MoAlB SCs is described in Fig. 4d. A synergistic effect is involved in the electrochemical reaction. Firstly, N_2_ is adsorbed and further accumulated on the surface of the MoAlB SCs by the strong binding between N and Al or B. Subsequently, with H^+^ absorbing and binding with N on the surface of MoAlB SCs, Mo acting as a catalytic site reduces N_2_ to NH_3_ gradually.

## Conclusions

In summary, MoAlB single crystals have been reported as a new candidate electrocatalyst for ambient-condition electrochemical ammonia synthesis and have demonstrated a high level of activity toward the electrochemical NRR in alkaline electrolytes. The as-synthesized MoAlB SCs afforded an NH_3_ yield of 9.2 µg h^−1^ cm^−2^ mg_cat._^−1^ and a Faradaic efficiency of 30.1% at − 0.05 V versus RHE. As revealed by the spectroscopic studies and electrochemical NRR tests, the outstanding NRR activity of MoAlB SCs was attributed to the synergistic role of Mo, Al and B atoms. Furthermore, mechanistic studies showed that MoAlB SCs possess facile NRR activity and selectivity due to their strong N_2_ adsorption and ability to overcome the competing hydrogen evolution reaction at reactive sites. The excellent catalytic performance and long-term stability of MoAlB SCs, combined with its convenient synthesis process, suggests that this system will be able to play a crucial role as a candidate pathway in the electrocatalytic NRR processes.

## Electronic supplementary material

Below is the link to the electronic supplementary material.

Supplementary material 1 (PDF 1935 kb)
