# Supplementary material for: Transition Metal Aluminum Boride as a New Candidate for Ambient-Condition Electrochemical Ammonia Synthesis
Source: Nanomicro Lett. 2020 Feb 28;12:65. doi: 10.1007/s40820-020-0400-z (PMC7770662; doi:10.1007/s40820-020-0400-z)
Supplement: Supplementary file 1 — Supplementary material 1 (PDF 1935 kb) [file 40820_2020_400_MOESM1_ESM.pdf]

Supporting Information for

Transition Metal Aluminum Boride as a New Candidate for Ambient-Condition Electrochemical Ammonia Synthesis

Yang Fu[1, †], Peter Richardson[2, †], Kangkang Li[3], Hai Yu[3], Bing Yu[4], Scott Donne[1], Erich Kisi[2], Tianyi Ma[1, *]

[1]Discipline of Chemistry, School of Environmental and Life Sciences, University of Newcastle, Callaghan, NSW 2308, Australia

[2]School of Engineering, University of Newcastle, Callaghan, NSW 2308, Australia

[3]CSIRO Energy, 10 Murray Dwyer Circuit, Mayfield West, NSW 2304, Australia

[4]School of Environmental Science and Engineering, Fujian Normal University, Fuzhou, Fujian 350007, People's Republic of China

[†] Yang Fu and Peter Richardson contributed eqully to this work

*Corresponding author. E-mail: Tianyi.Ma@newcastle.edu.au (Tianyi Ma)

S1 Material Characterization

Scanning electron microscopy (SEM) images were obtained on a Zeiss Sigma VP FESEM instrument operating at 3 kV after sputtering specimens with gold. Energy-dispersive X-ray spectroscopy (EDS) investigations were conducted using a Bruker EDS detector to gather information regarding weight% of elements present, their uniformity and co-ordination. Transmission electron microscopy (TEM) was conducted on a JEOL JEM-2100 HRTEM instrument operating at 200 kV, equipped with a JEOL JED-2300 EDS detector. The high-angle annular dark field scanning transmission electron microscopy (HAADF-STEM) images were recorded using a FEI Titan G2 80-300 microscope at 300 kV equipped with a probe corrector. The crystallinity and phase purity of obtained samples were characterized by X-ray diffraction (XRD) patterns, recorded on a Panalytical X'Pert™ diffractometer with Cu-Kα radiation. X-ray photoelectron spectroscopy (XPS) measurements were performed using a Thermo SCIENTIFIC K-Alpha+ X-ray photoelectron spectrometer. All electrochemical measurements were carried out on a CHI760e electrochemical station. Detection of ammonia was recorded using a spectrophotometer (UV-1800, SHIMADZU).

S2 Calculations

1) Ammonia yields were calculated using Eq. S1:

$$R_{NH_3}(\mu g \cdot cm^{-2} \cdot h^{-1}) = \frac{c\ (ppb) \times V(L)}{t(h) \times S(cm^{-2})} \qquad (S1)$$

Where:

$R_{NH_3}(\mu g \cdot cm^{-2} \cdot h^{-1})$: Ammonia formation yield

c (ppb): Ammonia concentration in the detection solution in ppb (μg/L)

V (L): Volume of solution in litres

t (h): Reaction time in hours

S: Active area of the membrane electrode in cm^{-2}

2) Faraday efficiency of ammonia was determined using Eq. S2:

$$FE(\%) = \frac{3 \times c\ (ppb) \times 10^{-6} \times V\ (L) \times F}{17 \times Q\ (C)} \times 100\ \% \qquad \text{(S2)}$$

Where:

F: Faraday constant in C/mol;

Q: Total quantity of electric charge in C.

S3 Detection of Ammonia

Low ammonia concentration detection was carried out by spectrophotometry (indophenol blue method) and/or ion selective electrode analysis (Orion™ High-Performance Ammonia Electrode 9512HPBNWP) methods. In addition, only a single absorber was used in the experiments.

S3.1 Ammonia-selective Electrode Method

Apparatus: Ammonia ion selective electrode – Ionic Strength Adjuster (Thermo Scientific Orion high performance ammonia ion selective electrode Cat.No.9512HPBNWP); Electrometer.

Reagents: Ammonia-free water; Ammonia standard solution: 1000 ppm ammonium chloride as Nitrogen standard (NH^{4+}); Low level Ammonia pH-adjusting Ionic Strength Adjuster (ISA).

Procedures:

a) Preparation of ammonia standards: A series of standard solutions were prepared with the concentrations of 20, 200, and 2000 ppb (NH^{4+}) in 0.1 M KOH (absorber).

b) Electrometer calibration: the following steps were performed during the electrometer calibration:

   i. The electrode was soaked in an ammonia electrode storage solution for at least 15 min.

   ii. The meter measurement mode was operated in mV mode.

   iii. 100 mL of each standard was measured into separate, clean beakers. 2 mL of Low level Ammonia pH-adjusting Ionic Strength Adjuster was added to each beaker prior to calibration, followed by waiting until the reading was stable for 2 min.

iv. The mV and ppb values were used as the Y axis and X axis respectively, to prepare the standard curve.

v. The electrode slope was checked for validity (slope should be between 54 and 60 in a temperature range of 20-25 °C).

c) 50 mL of sample was measured and 1 mL of Low level Ammonia pH-adjusting Ionic Strength Adjuster was added. When getting readings which were stable for at least 2 min, the measurement was recorded.

d) Calculation

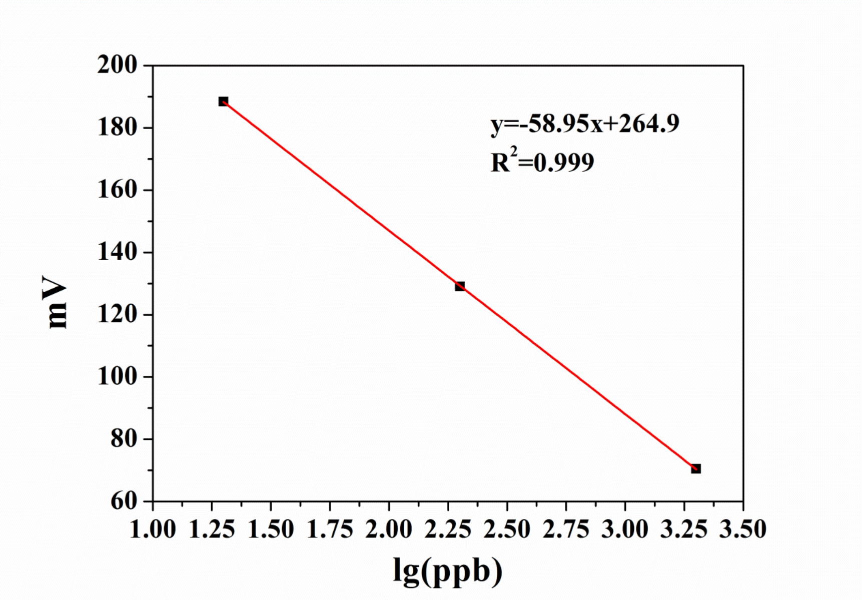

Fig. S1 Calibration of NH_3 in 0.1M KOH from 20 ppb to 2000 ppb

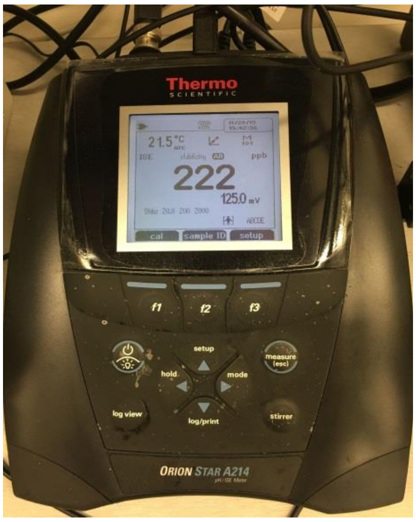

Fig. S2 Photograph of ammonia-sensitive testing instrument

S3.2 Indophenol Blue Method

In addition, compared to the Ammonia-selective electrode method, a colorimetric method using

indophenol blue for NH_3 detection was also performed to confirm the reliability of the former method.

Apparatus: A spectrophotometer (UV-1800, SHIMADZU) was used at fixed wavelength (λ=630 nm).

Reagents: Phenol (BDH Laboratory Supplies), ammonium sulphate (BDH Chemical, Australia Pty. Ltd.), ethanol (Merck KGaA), sodium nitroferricyanide (III) dehydrate (Sigma-Aldrich, > 99 %), trisodium citrate dehydrate (Sigma-Aldrich), sodium hydroxide (Sigma-Aldrich), sodium hypochlorite solution (Sigma-Aldrich).

Procedures:

a) Preparation of special reagents:

i. Phenol-alcohol reagent: Dissolve 10 g of phenol in 95% ethyl alcohol to a final volume of 100 ml.

ii. Sodium nitroprusside (nitroferricyanide): Dissolve 1 g in DI water to a final volume of 200 ml. Store in dark bottle for no more than 1 month.

iii. Alkaline complexing reagent: Dissolve 100 g of trisodium citrate and 5 g of sodium hydroxide in DI water to a final volume of 500 ml.

iv. Oxidizing solution: Add 100 ml alkaline complexing reagent to 25 ml sodium hypochlorite (as fresh as possible).

b) Measurement:

i. Preparation of NH_4-N standards: A series of NH4-N standard solutions were prepared with the concentrations of 0, 50, 100, 200, 500, 750, and 1000 ppb (NH_4^+) by dissolving in $(NH_4)_2SO_4$.

ii. 10 mL of standard or sample solution was taken. Then 400 μL of phenol solution, 400 μL of nitroferricyanide solution and 1 ml oxidizing reagent were added respectively to the standard or sample solution. Absorbance measurements of standards and unknown samples were performed at λ=630 nm using a spectrophotometer after mixing the solutions well for at least 1 h.

iii. Absorbance values of standards were used to generate a standard curve. The standard curve below was used to calculate the ammonia concentration in unknown solutions.

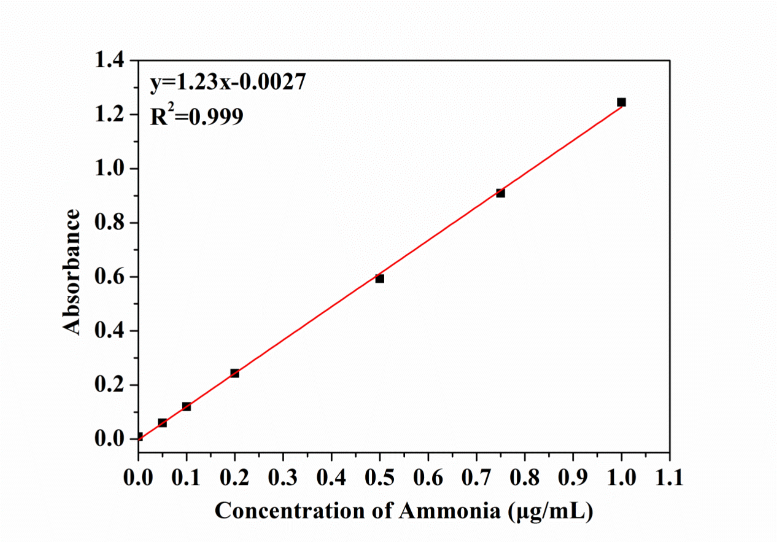

Fig. S3 Calibration curve for colorimetric NH_3 assay using the Indophenol blue method

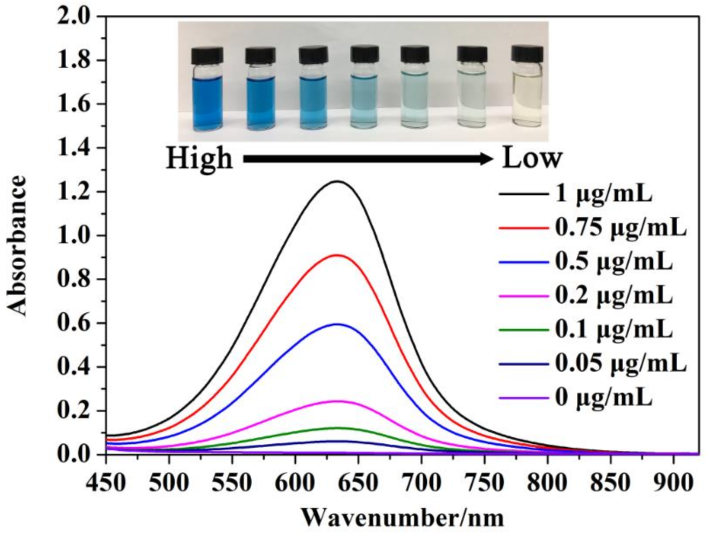

Fig. S4 UV-vis curves of various NH_3 concentration based on Indophenol blue method, and the insert shows the chromogenic reaction of Indophenol blue indicator with NH_3

S3.3 Determination of Hydrazine Hydrate

In addition, due to existing by-products, the yield of hydrazine in the electrolyte was examined by the method of Watt and Chrisp [S1]. A color reagent was prepared by a mixture of para-(dimethylamino) benzaldehyde (5.99 g), HCl (concentrated, 30 mL) and ethanol (300 mL). A calibration curve was plotted as follows: First, preparing a series of standard solutions with the concentrations of 0, 50, 100, 200, and 500 ppb by pipetting suitable volumes of the hydrazine hydrate-nitrogen 0.1 M KOH solution. Then, the absorbance of the standard or sample solution was measured at λ=460 nm after mixing 5 mL of standard or sample solution with 5 mL of color reagent. Finally, the yields of hydrazine in unknown sample solutions were estimated from a standard curve.

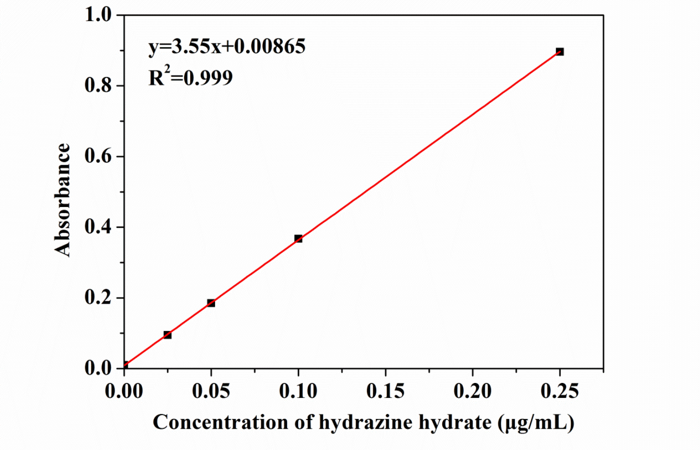

Fig. S5 Calibration curve for the colorimetric $N_2H_4 \cdot H_2O$ assay using the method of Watt and Chrisp

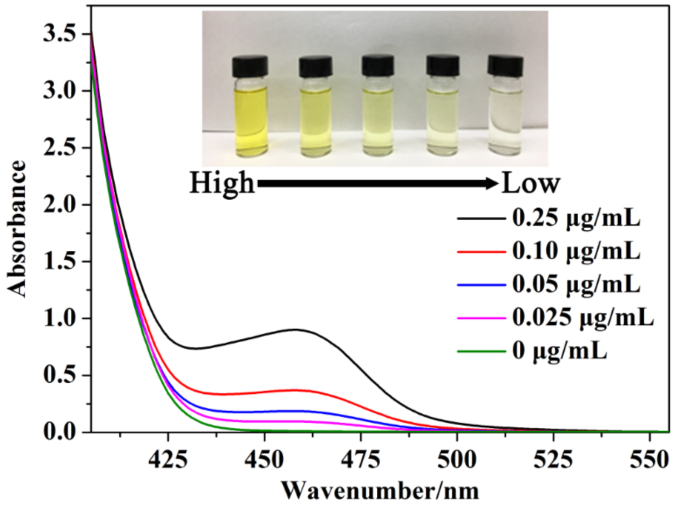

Fig. S6 UV-vis curves of various N_2H_4 concentrations, and the insert shows the chromogenic reaction of para-dimethylamino-benzaldehyde indicator with N_2H_4

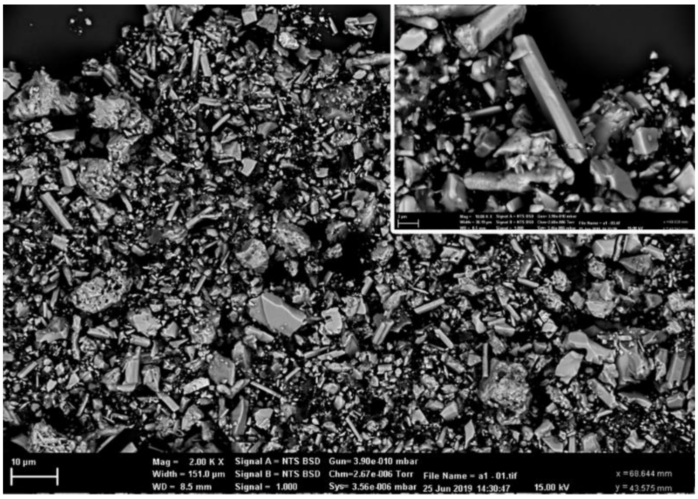

Fig. S7 SEM images of MoAlB SCs

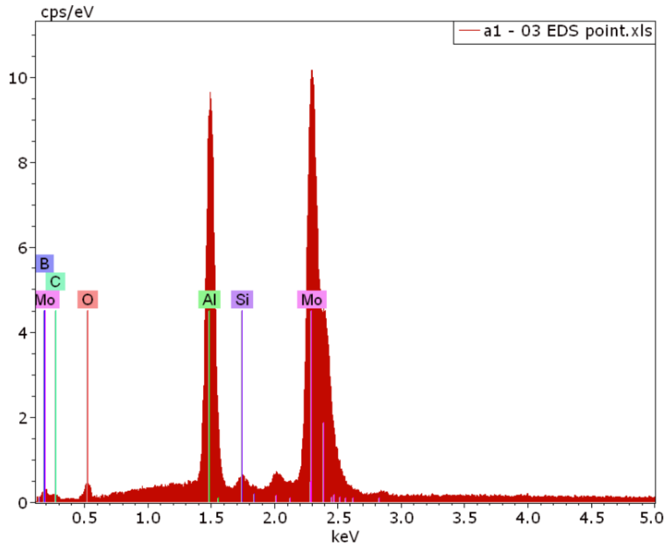

Fig. S8 EDS point analysis of the MoAlB SCs

Table S1 The amounts of elements atom and weight by EDS

**Element**	**series**	**wt%**	**at%**
Boron	K-series	7.258457928	25.9421762
Carbon	K-series	2.658793147	8.553291868
Oxygen	K-series	4.317931307	10.42797402
Aluminium	K-series	19.39037932	27.76818405
Silicon	K-series	0.592503425	0.815148274
Molybdenum	L-series	65.78193487	26.49322558
	Sum:	100	100

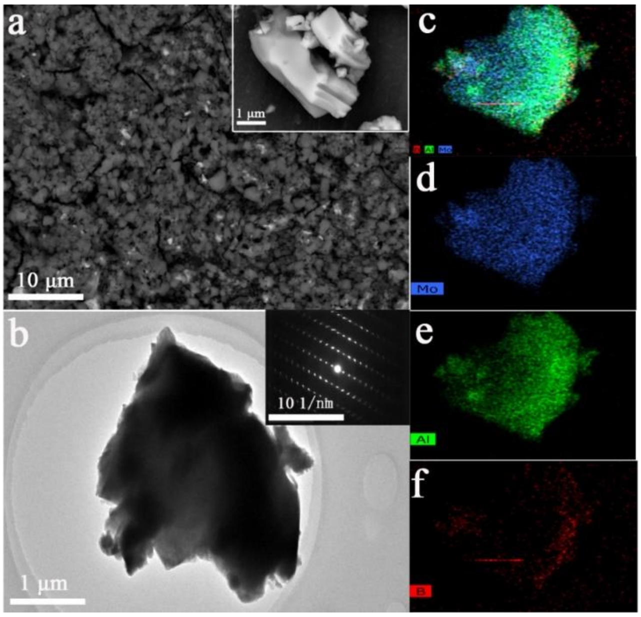

Fig. S9 a SEM image of bulk MoAlB powder; **b** TEM image of a bulk MoAlB particle, the insert shows the associated SAED pattern; **c-f** EDS elemental mapping results for the bulk MoAlB particle in **b**

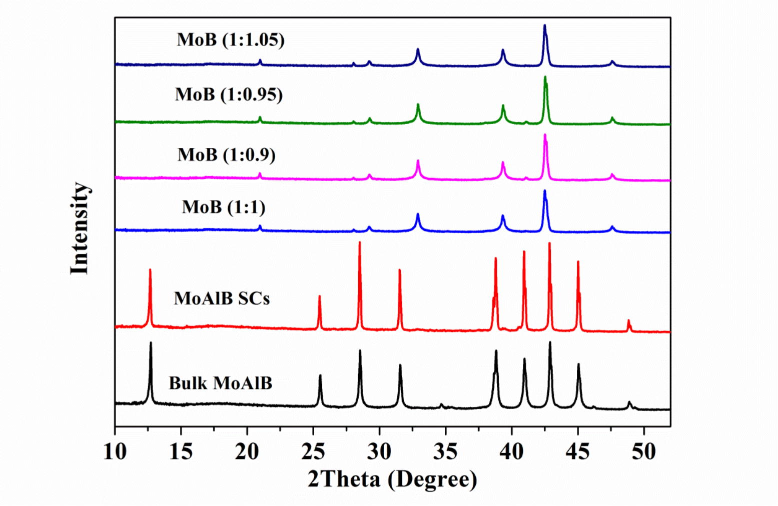

Fig. S10 XRD patterns of bulk MoAlB, MoAlB SCs, MoB (1:1), MoB (1:0.9), MoB (1:0.95), and MoB (1: 1.05)

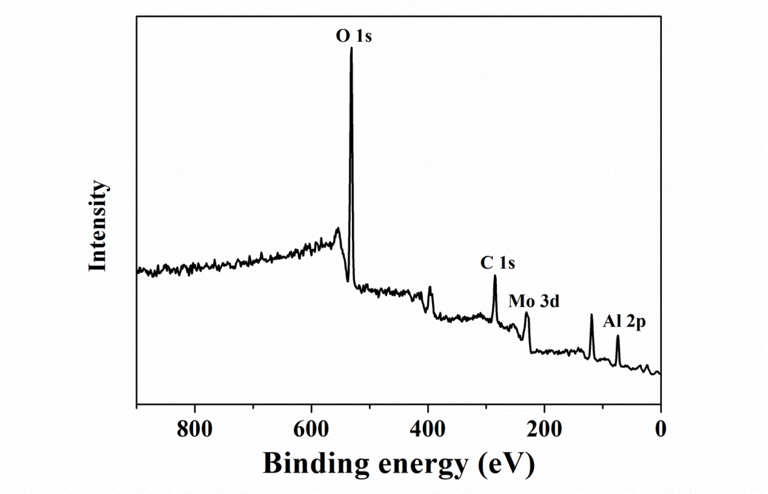

Fig. S11 XPS spectrum for MoAlB SCs

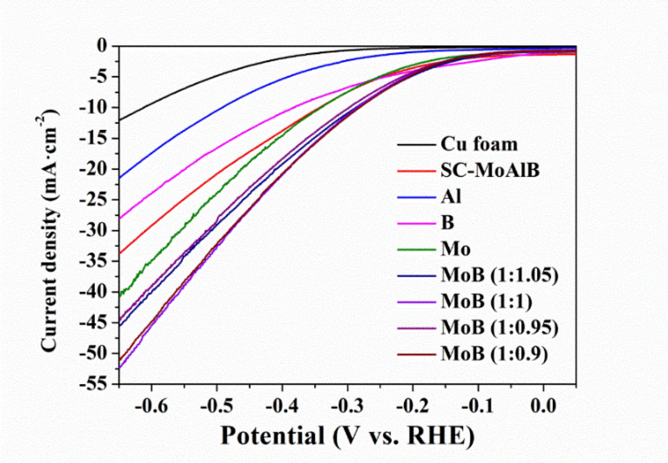

Fig. S12 LSV curves of pure Cu foam, MoAlB SCs, Al, B, Mo, MoB (1: 1.05), MoB (1: 1), MoB (1: 0.95), and MoB (1: 0.9) electrodes in an N_2-saturated aqueous solution of 0.1 M KOH

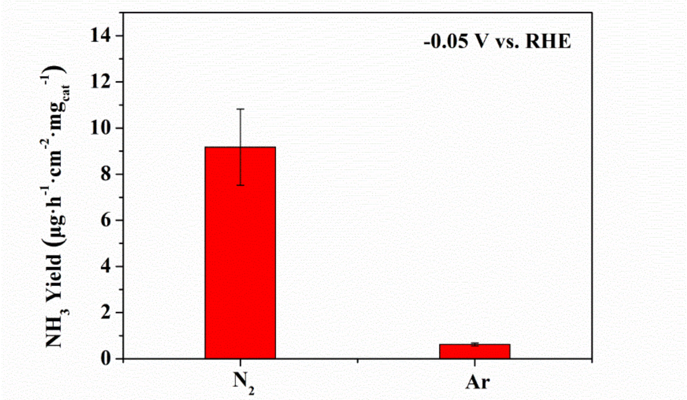

Fig. S13 Ammonia yields for the MoAlB SCs/Cu foam electrode in an N_2 and Ar-saturated aqueous solution of 0.1 M KOH

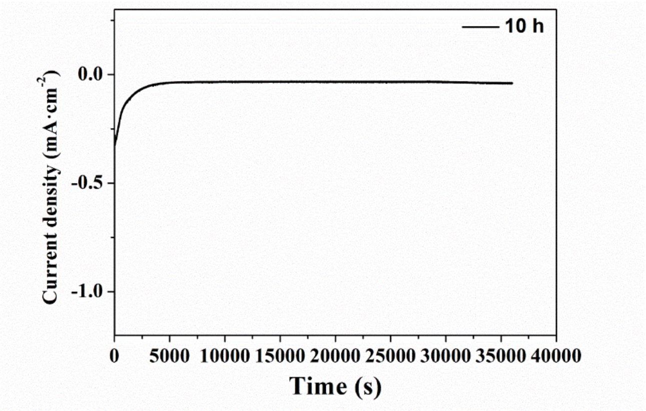

Fig. S14 Chronoamperometry results at the -0.05 V vs. RHE within 10 h test

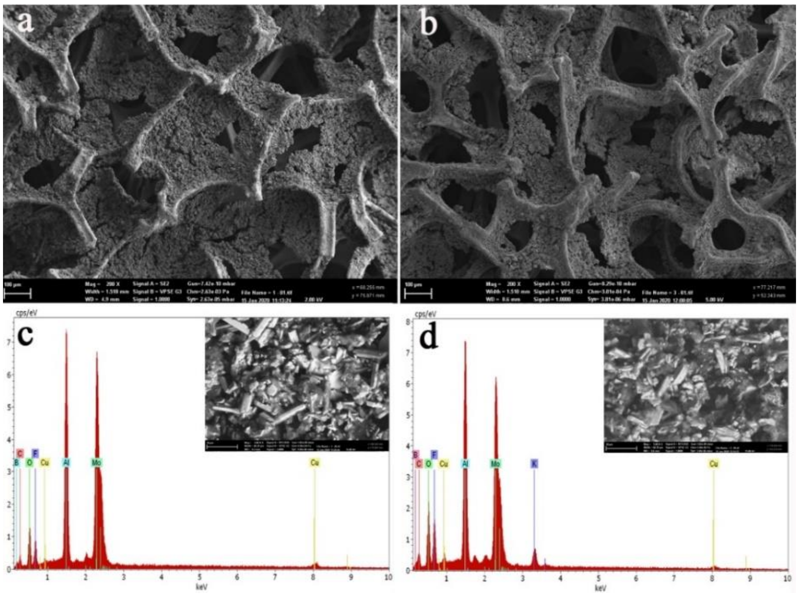

Fig. S15 a SEM image of MoAlB SCs/Cu foam before NRR stability tests; **b** SEM image of MoAlB SCs/Cu foam after NRR stability tests; **c** EDS region analysis of the MoAlB SCs/Cu foam before NRR stability tests; **d** EDS region analysis of the MoAlB SCs/Cu foam before NRR stability tests; The insert shows the corresponding SEM image of EDS region scan

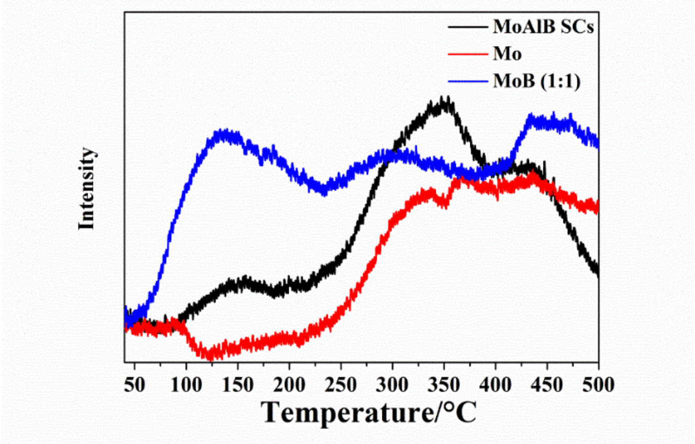

Fig. S16 The N_2-TPD of MoAlB SCs, Mo and MoB (1:1)

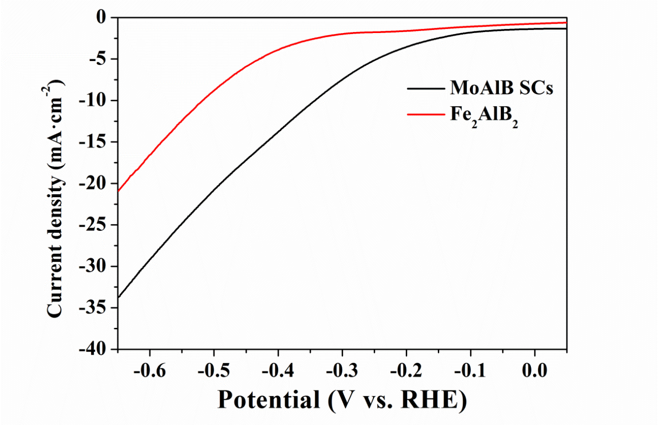

Fig. S17 LSV curves of MoAlB SCs and Fe_2AlB_2 electrode in an N_2-saturated aqueous solution of 0.1 M KOH

Table S2 A brief summary of the representative reports on electrochemical N_2 reduction in aqueous solutions at ambient conditions

Temperature	Catalyst	Electrolyte	Yield	FE (%)	Potential	Refs.
25 °C	Au nanorod	0.1 M KOH	1.648 $\mu g \cdot h^{-1} \cdot cm^{-2}$	4.02	-0.2 V vs. RHE	[S2]
25 °C	a-Au/CeO_x-RGO	0.1 M HCl	8.3 $\mu g \cdot h^{-1} \cdot mg^{-1}$	10.1	-0.2 V vs. RHE	[S3]
R.T.	30 wt% Fe_2O_3–CNT	0.50 M KOH	0.649 $\mu g \cdot h^{-1} \cdot cm^{-2}$	0.164	-2.0 V vs Ag/AgCl	[S4]
R.T.	VN (Nanowires)/CC	0.1 M HCl	2.48×10^{-10} mol s^{-1} cm^{-2}	3.58	-0.3 V vs RHE	[S5]
25 °C	ZIF-derived disordered carbon	0.1 M KOH	9.22 mmol g^{-1} h^{-1}	10.2	-0.3 V vs RHE	[S6]
25 °C	B-doped graphene	0.05 M H_2SO_4	9.8 μg cm^{-2} h^{-1}	10.8	-0.5 V vs RHE	[S7]
R.T.	Single Mo atoms anchored on N-	0.1M KOH	34.0 mg h^{-1} mg_{cat}^{-1}	14.6	-0.3 V vs. RHE	[S8]

	doped porous carbon					
R.T.	Boron nanosheet	0.1 M Na_2SO_4	13.22 μg h^{-1}	4.04	-0.80 V vs, RHE	[S9]
R.T.	MoAlB SCs	0.1 M KOH	9.2 μg h^{-1} cm^{-2} $mg_{cat.}^{-1}$	30.1	-0.05 V vs. RHE	This work

Table S3 Amounts of element atoms and weight by EDS region scan before and after NRR stability tests

	**Element**	**series**	**wt%**	**at%**
Before	Boron	K-series	3.734440272	10.5929913
	Carbon	K-series	7.010116388	17.8980216
	Oxygen	K-series	13.39565904	25.67552637
	Fluorine	K-series	5.869979419	9.474984723
	Aluminium	K-series	16.37436737	18.61044523
	Copper	K-series	3.746501132	1.807992468
	Molybdenum	L-series	49.86893638	15.94003823
		Sum:	100	100
After	Boron	K-series	5.654867256	13.46861
	Carbon	K-series	6.149088269	13.1825
	Oxygen	K-series	18.27592052	29.41318
	Fluorine	K-series	10.76570021	14.59122
	Aluminium	K-series	16.71550044	15.95214
	Potassium	K-series	4.024072383	2.650173
	Copper	K-series	3.157483003	1.279438
	Molybdenum	L-series	35.25736793	9.462732
		Sum:	100	100

Supplementary References

[S1] G.W. Watt, J.D. Chrisp, Spectrohotometric method for the determination of hydrazine. Anal. Chem. **24**, 2006–2008 (1952). https://doi.org/10.1021/ac60072a044

[S2] D. Bao, Q. Zhang, F.L. Meng, H. X. Zhong, M.M. Shi et al., Electrochemical reduction of N_2 under ambient conditions for artificial N_2 fixation and renewable energy storage using N_2/NH_3 cycle. Adv. Mater. **29**, 1604799 (2017). https://doi.org/10.1002/adma.201604799

[S3] S.J. Li, D. Bao, M.M. Shi, B.R. Wulan, J.M. Yan, Q. Jiang, Amorphizing of Au nanoparticles by CeOx-RGO hybrid support towards highly efficient electrocatalyst for N_2 reduction under ambient conditions. Adv. Mater. **29**, 1700001 (2017). https://doi.org/10.1002/adma.201700001

[S4] S. Chen, S. Perathoner, C. Ampelli, C. Mebrahtu, D. Su, G. Centi, Room-temperature electrocatalytic synthesis of NH_3 from H_2O and N_2 in a gas–liquid–solid three-phase reactor. ACS Sustain. Chem. Eng. **5**, 7393 (2017). https://doi.org/10.1021/acssuschemeng.7b01742

[S5] X. Zhang, R. Kong, H. Du, L. Xia, F. Qu, Highly efficient electrochemical ammonia synthesis vianitrogen reduction reactions on a VN nanowire array under ambient conditions. Chem. Commun. **54**, 5323–5325 (2018). https://doi.org/10.1039/c8cc00459e

[S6] S. Mukherjeea, D.A. Cullenb, S. Karakalosc, K. Liud, H. Zhanga et al., Metal-organic framework-derived nitrogen-doped highly disordered carbon for electrochemical ammonia synthesis using N_2 and H_2O in alkaline electrolytes. Nano Energy **48**, 217 (2018). https://doi.org/10.1016/j.nanoen.2018.03.059

[S7] X. Yu, P. Han, Z. Wei, L. Huang, Z. Gu, S. Peng, J. Ma, G. Zheng, Boron-doped graphene for electrocatalytic N_2 reduction. Joule **2**, 1610 (2018). https://doi.org/10.1016/j.joule.2018.06.007

[S8] L.L. Han, X.J. Liu, J.P. Chen, R. Q. Lin, H.X. Liu et al., Atomically dispersed molybdenum catalysts for efficient ambient nitrogen Fixation. Angew. Chem. Int. Ed. **58**, 2321–2325 (2019). https://doi.org/10.1002/anie.201811728

[S9] X.X. Zhang, T.W. Wu, H.B. Wang, R.B. Zhao, H.Y. Chen et al., Boron Nanosheet: An elemental two-dimensional (2D), material for ambient electrocatalytic N_2-to-NH_3 fixation in neutral media. ACS Catal. **9**, 4609–4615 (2019). https://doi.org/10.1021/acscatal.8b05134
